# Cases in Hospital Practice

**Published:** 1846-03

**Authors:** Jonathan Toogood


*Cases in Hospital Practice.* By Jonathan Toogood, M.D., F.R.C.S.

*Case* I. *Amputation below the Knee: Secondary Hæmorrhage.*—John Mosse, of Woolavington, was admitted into the Bridgewater Infirmary for disease of the foot, for which the leg was amputated at the usual place, below the knee, on Tuesday. The stump was opened on the Friday following, when the whole surface was found in a state of sphacelus. The sloughs, which were superficial, began to separate the next day, in the afternoon of which some hemorrhage took place. No vessel could be detected and it soon ceased, but returned again on the following morning, when a most minute examination of the stump was made, but no vessel could be discovered; the whole surface was in a soft pulpy state. Quiet and cold applications were directed, and no further bleeding occurred that day; but at twelve at night an alarming discharge of blood took place. As the patient was much sunk, it was determined to tie the femoral artery in front of the thigh; after this he lost no more blood, the stump soon assumed a healthy appearance, and healed in the usual way.

*Case* II. *Amputation above the Knee: Secondary Hæmorrhage.*—A young woman was admitted on account of a disease of the knee-joint, of many years standing, which had greatly reduced her strength. The thigh was amputated in the usual way, and she went on favourably for ten days, when considerable hæmorrhage took place, which was arrested by cold; it recurred two days afterwards, when a vessel was tied, but the bleeding returning several times, although to no great extent, weakened her so much that it was considered advisable to tie the femoral artery as high up as possible. This was effected without difficulty, after which there was no interruption to her recovery.

*Case* III. *Extensive Burn: Amputation at the Shoulder-Joint.*—James Ellis, aged 22, fell into a large fire on the hearth during an epileptic fit, and being alone, was not discovered until the right arm was burnt from the fingers to the shoulder, and the left to the wrist, some parts of each to a cinder. The abdomen, penis, breast, and back, were also extensively burnt. He was dressed, and removed the next day to the Infirmary. He refused to submit to amputation, but consented at the end of a week. There was scarcely room to perform the operation, and no chance of covering the stump, the integuments and muscles were burnt so close to the joint; besides which, the other injuries were so extensive, as to render the case very hazardous and unpromising. The artery was compressed with the thumb above the clavicle, and he lost but little blood, but it was necessary to tie several small arteries. He went on tolerably well, but on opening the stump on the fourth day, the whole surface presented a tawny sloughing appearance. It was dressed twice a day with red precipitate ointment, and bark, wine, and opium were liberally given. On the fifth morning after the operation, the left hand was removed above the wrist, after which the wound of the shoulder improved daily, and in seven weeks all the wounds were healed. For the next seven weeks he had no fit, but after that time the fits returned as frequently as ever. He lived for many years, and at last died suddenly.

Two other cases have occurred during my practice, and although the operation succeeded in both for a time, the patients eventually died from disease of the lungs.

*Case* IV. *Carcinoma of the Breast: Operation.*—Numerous cases of disease of the breast have been presented to my notice, and amongst them many of a truly carcinomatous character. I have been frequently called on to remove them with the knife, and have often assisted others; but I never remember a case which terminated successfully, although in some instances the operation has been undertaken under the most favourable circumstances. The event of the following case determined me never to advise the operation, although I would not refuse to perform it at the desire of the patient, after explaining fully the doubtful results.

A strong healthy woman, aged 50, had a large carcinomatous tumour in the breast, for which she had consulted several practitioners of eminence, all of whom agreed, as there was no appearance of the glands in the axilla or above the clavicle being diseased, in the propriety of the operation. I removed the whole breast from the pectoral muscle, and as no ligature was required, the parts were evenly brought together and healed in ten days completely. No constitutional disturbance followed, and never did a case promise a more successful termination. She continued perfectly well to the end of six weeks, when I was requested to see her on account of some uneasiness in the course of the cicatrix, which was attributed to the friction of her stays. On examination I observed a small pimple on the base of the cicatrix, which soon increased and became a troublesome sore, which rapidly spread, destroying all the parts around, and attacking the other breast. Her state soon became deplorable, and in less than six months she died.—***Prov. Med. and Surg. Journ.***

